# Youth, the New Adolescence: A Challenge and a Window of Opportunity for Early Mental Health Interventions

**DOI:** 10.32872/cpe.16951

**Published:** 2025-02-28

**Authors:** Simone Munsch, Tina In-Albon, Nadine Messerli-Bürgy

**Affiliations:** 1 Department of Psychology, Clinical Psychology and Psychotherapy, University of Fribourg, Fribourg, Switzerland; 2 Food Research and Innovation Center, FRIC, Cluster Food and Mental Health / Psychology, University of Fribourg, Fribourg, Switzerland; 3 Clinical Child and Adolescent Psychology and Psychotherapy, University of Mannheim, Mannheim, Germany; 4 Family and Development Research Center, Institute of Psychology, University of Lausanne, Lausanne, Switzerland

At the beginning of 2025, we invite you to focus on some of the mental health challenges that lie ahead. Our attention is focused on youth, particularly those between the ages of 12 and 25 (Insel & Fenton, 2005), a period often referred to as the "new adolescence" (Sawyer et al., 2018). While there is evidence on an earlier onset of puberty and on continued growth well into the 20s, there is also knowledge on a delayed timing of role transitions, including the completion of education and parenthood which let to the new conceptualization of this period as the new adolescence (Sawyer et al., 2018). It is also essential to recognize that despite advances in psychotherapy, in neuroscience and psychopharmacological approaches, there are no quick solutions to stop or slow down the global increase of mental health problems in youth. In recent years, this increase has been particularly evident among children and adolescents, driven by factors such as uncertainty, crises, and conflicts in our world.

However, we all wish childhood and adolescence to be a period of positive development that is, at times, maybe feeling shaky but happy, with most young people overcoming perceived challenges and growing out of any problems. While this is still the case for the majority of children, adolescents and young adults, we cannot ignore the fact that the peak age of onset of mental disorders in the general population is around 5.5 and 15.5 years for anxiety disorders, 14.5 years for obsessive-compulsive disorder, 15.5 years for eating disorders, 19.5 years for substance use disorders, 20.5 years for schizophrenia/psychotic disorders and personality disorders, and 20.5 years for mood disorders (Solmi et al., 2022). In other words, by the age of 24 years, 75% of all mental disorders an individual might experience in his life had already occurred for the first time (Kessler et al., 2001).

Why should the period of adolescence be expanded to ‘new adolescence’? This idea is not a TikTok move or of any other social media, but an evidence-based strategy to address the challenges of today’s world of children, adolescents and young adults and besides this, not to let windows of opportunity go unused that allow early intervention and prevention improving mental health. Therefore, youth is not only a critical period but a period of meaningful development that allows long-term changes in an individual’s functioning. Does that get you excited? We fully understand that for you—the active members of this community —enthusiasm alone is not enough, but independently of your specialization in child, adolescent, or adult mental health, you probably agree that reliable data is needed to show that this period is not only critical for emotional, social, cognitive and occupational development (Thompson et al., 2023), but that it can be influenced in a meaningful way.

Let’s start with the term “window of opportunity” which is a sensitive period in the child’ and adolescent’s development of neurobiological systems such as systems related to threat, reward, social cognition and stress and generally the formation of multiple complex neural systems enabling an individual to live through, and therefore perceive and process a given situation in the context of multiple, at best, coping-oriented experiences and neurobiological systems.

Research shows that in addition to data on the importance of youth for the development of mental disorders (Solmi et al., 2022), we also have data on the sensitivity of the underlying biological systems up to the age of 25 years (Uhlhaas et al., 2023). Although this is good news, it is not enough for us as psychologists because we all know that youth is a time of more complex interplay of different factors. Besides the impact of changes in parent-child and peer interactions, the adolescent’s living context (degree of urbanization, economic background, access to education) but also the levels of stigmatization of mental health problems within adolescent’s environment and limited access to mental health support all impact on an adolescent’s mental wellbeing (Orben et al., 2020; Speyer et al., 2022; van der Wal et al., 2021). It’s up to us to highlight the importance of this developmental window and mobilize ourselves, our colleagues, and policymakers to support and improve mental health in this critical period of youth.

While some aspects of today's 12-25-year-olds’ daily lives are new to us, we must acknowledge that a person’s integration into society has long been recognized as a developmental period (Erikson, 1959). Let’s face it. The idea that adolescence doesn’t neatly end at the age of 18 has been around for decades and is sometimes consistent with our own “lived experience”. Yet, despite this, mental health systems, diagnostics and research efforts worldwide have been slow to fully reflect this understanding and consider it consistently in diagnostics, treatment and prevention.

We do not want to waste precious time in the early year of 2025, and we therefore call on you to contribute to the idea of making the most of these windows of opportunity in your daily research and clinical work, which means:

- **Broaden the age range of your research**: Include experts who are skilled in adapting questionnaires, clinical interviews, and interventions for this age group. Even if "youth" ranges from 12 to 25, it’s important to recognize that instruments need to be adapted because cognitive and emotional development limit the assessment of youth.
- **Broaden your treatment approach**: When treating children, adolescents and young adults with or at risk for mental health problems, use your expertise in developmental psychopathology to anticipate future needs. Ask yourself: What will this child’s mental health look like at the age of 16? Where should we intervene to support their well-being at the age of 22? How might the family system be able to adapt to the child or young person’s needs and promote positive change?
- **Educate parents**: Help parents to better understand their evolving role in the modern world and provide parental training to help them model resilience, cope with daily challenges, and promote mental well-being in their children.
- **Advocate for mental health policies**: Advocate for policies that view youth mental health as a critical window of opportunity, which should be recognized and promoted wherever and whenever possible.
- **Include youth in your research**: Youth with lived experience or youth in the same age range as your research samples can provide valuable feedback and help to advance the understanding of mental health problems in youth.

We thank you for your patience if you find our enthusiasm is too strong at the beginning of this year. However, we have long been committed to contributing to the youth mental health paradigm and would like to do so more this year in our new role as editor-in-chief and editors of CPE. We look forward to your contributions and to the exchange with you on this topic.

We thank the publishers and the reviewers for their excellent contribution to open science and look forward to the upcoming CPE volumes with the same enthusiasm. We thank you for your critical commitment and for including a developmental psychology perspective in your research and clinical work.
